# The frequency of pulmonary hypertension in newborn with intrauterine growth restriction

**DOI:** 10.1038/s41598-020-65065-2

**Published:** 2020-05-15

**Authors:** Ghulam Abbas, Shahid Shah, Muhammad Hanif, Abid Shah, Anees ur Rehman, Sana Tahir, Komal Nayab, Arzoo Asghar

**Affiliations:** 1 Department of Pharmaceutics, Faculty of Pharmaceutical Sciences Government College University Faisalabad, Faisalabad, Pakistan; 2 Department of Pharmacy Practice, Faculty of Pharmaceutical Sciences Government College University Faisalabad, Faisalabad, Pakistan; 3 Department of Pharmaceutics, Faculty of Pharmacy, Bahauddin Zakariya University Multan, Multan, Pakistan; 4 Children hospital and the Institute of Child Health Multan, Multan, Pakistan; 5 Department of Clinical Pharmacy, School of Pharmaceutical Sciences, University Sains Penang, Sains Penang, Malaysia

**Keywords:** Intrauterine growth, Paediatric research

## Abstract

Intrauterine growth restriction (IUGR) is a clinical definition applied to neonates born with clinical features of malnutrition and in-utero growth retardation irrespective of their birth weight percentile. This study was aimed to determine the frequency of pulmonary hypertension (PH) in neonates with IUGR. In this descriptive cross-sectional study, we followed 96 neonates with IUGR (≤28 days) and 38 neonates without IUGR born in the department of the neonatal intensive care unit children hospital complex Multan, Pakistan. We analyzed certain factors such as gender, gestational age (GA) (weeks), birth weight (BW in kg), weight percentile (WP) for GA, meconium aspiration syndrome (MAS), birth asphyxia (BA) and respiratory distress syndrome (RDS) for pulmonary hypertension (PH) in IUGR and non-IUGR group. GA was measured by the Ballard scoring system. Echocardiography was performed for all patients by the pediatric cardiologist to measure pulmonary arterial (PA) pressure using Bernoulli’s equation. Out of total 96 IUGR neonates, 33.3% (n = 32) suffered from PH, of which 65.3% (n = 18) were male and 43.7% (n = 14) were female. The percentages of IUGR neonates with BA, MAS and RDS were 34.4%, 18.8% and 22.9% respectively. The data were analyzed using the SPSS-16 software to test the statistical significance of the results. A *p*-value less than 0.05 was considered significant. When the chi-square test was applied, it depicted that MAS was significantly associated with PH in IUGR neonates (*p* = *0.0001*) compared to non-IUGR neonates. Our findings suggested an increased chance of PH in IUGR neonates and MAS may be a strong factor.

## Introduction

Intrauterine growth restriction (IUGR) is an increasingly recognized problem that affects 7 to 15% of pregnancies worldwide^1^. IUGR fetuses experiencing growth restriction inside the uterus fails to achieve their full growth potential for a given gestational age (GA in weeks) and are at increased risk of perinatal mortality and morbidity^2,3^. The reduced rate of fetal growth in IUGR is essentially an adaptation to an unfavorable intrauterine environment and it can result in enduring modifications in metabolism, growth, and development^4–6^. IUGR occurs in small for gestational age (SGA) neonates with birth weight (BW in kg) <10 percentiles^3,7,8^. About 10% of all live born babies and at least 30% of the neonates having low birth weight (LBW: <2.5 kg irrespective of gestational age^9^) suffer from IUGR^10^. The perinatal mortality in IUGR neonates is 4 to 10 times higher than that of normal grown babies^11^.

Although the etiology of IUGR is variable,^2^ however, 80 to 90% of all cases of IUGR liable to preventive and therapeutic management involves an impaired transplacental supply of oxygen and nutrients to the fetus^3^. IUGR is the hallmark of fetal hypoxia^12^. IUGR induced by an adverse intrauterine environment, such as uteroplacental vascular insufficiency and maternal malnutrition, results in varying degree of pulmonary vascular remodeling^11^, which leads to pulmonary hypertension (PH)^13,14^.

Poor growth also exposes the fetus to perinatal and neonatal complications like meconium aspiration syndrome (MAS), birth asphyxia (BA) and respiratory distress syndrome (RDS)^7,15^. MAS is commonly an intrauterine event that occurs in response to a hypoxic environment^16^. MAS has been commonly associated with PH because the direct lungs damage from meconium^17^ ends with mechanical obstruction of airways^18^, inactivation of surfactants^19^ and constriction of the pulmonary vessels^20^. IUGR fetuses with chronic intrauterine hypoxia are also prone to asphyxia^21^ because in the asphyxiated neonates hypoxia increases pulmonary vascular resistance which results in PH^17^. Hypoxia induced IUGR neonates with a mild form of RDS may also experience an acute episode of PH^11^.

The incidence of IUGR in developing countries is 6 times higher as compared to developed countries. In developing countries, the incidence of IUGR was reported to be 11% of the total number of neonates in 2008^22^, but in 2017, it was up to 30% that constituted 50 to 60% of LBW^23^.

Preventing LBW infants in developing countries should be considered as a crucial public health priority^24,25^, where the condition is essentially attributed to IUGR. As Pakistan is the developing country, the incidence of mortalities and injuries caused by IUGR associated PH is higher. In this scenario, it is necessary to reduce the burden of IUGR linked PH. We have conducted this study for the first time in Pakistan to shed light on the incidence of PH in IUGR neonates. The aim of this study was to determine the incidence of PH in IUGR neonates.

## Materials and Methods

### Study design

The study design was descriptive cross-sectional.

### Setting

The data was collected within the department of the neonatal intensive care unit of the children hospital complex Multan, Pakistan during 1^st^ July 2018 to 30^th^ June 2019.

### Ethical approval

The study protocol has received ethical approval from the Government College University Faisalabad, Pakistan, Institutional Review Committee and the Health Research Committee of Children Hospital and the Institute of Child Health (ICH), Multan, Pakistan (# 009135). All methods were performed in accordance with the relevant guidelines and regulations. An informed consent from a parent and/or legal guardian was obtained.

### Participants

A non-probability consecutive sampling technique was used. The study was carried out in strict compliance and all procedures and protocols were approved by the ethical committee of the institution. The selected patients were subjected to take information by clinical examination with particular emphasis on the BW (kg) for GA and X-ray chest obtained in all neonates. GA (weeks) was measured by the Ballard scoring system. The reference values of weight for GA were compared to the growth chart. Echocardiography was performed for all patients within the first 28 days of life by the pediatric cardiologist to measure pulmonary arterial (PA) pressure using Bernoulli’s equation. This was done by combining tricuspid regurgitation (TR) jet pressure and right atrial pressure. In the absence of right ventricular outflow tract obstruction, right arterial pressure was considered as 10 mmHg. All newborns were managed according to the protocols designed for PH. PH was labeled if the pulmonary artery pressure was greater than 25 mmHg after exclusion of the neonates mentioned in the exclusion criteria.

### Inclusion criteria

Neonates (0-28 days) of both genders including pre-term (<37 weeks) and term (≥37 weeks) with BW (kg) less than the 10^th^ percentile of expected weight for GA, according to the WHO provided growth charts, were included in the study. In this study, 134 neonates were eligible after screening of 165 neonates. Out of 134 neonates, 96 were recruited with IUGR as a sample group (Group A) and 38 were non-IUGR as a references group (Group B). The incidence of PH was observed in 32 and 24 neonates from group A and B respectively.

### Exclusion criteria

Neonates with no or incomplete Doppler envelopes or congenital heart disease (detected by echocardiography), congenital diaphragmatic hernia (detected by CXR) and Down’s syndrome or other syndrome were excluded from this study.

### Statistical methods

The data were analyzed using the SPSS-16 statistical software. The descriptive statistics were applied to obtain a quantitative summary of parameters. The quantitative variable weight percentile (WP) for GA was calculated as the mean standard deviation. The effect modifier was controlled by stratification of gender, GA (weeks), and WP, presence or absence of BA, MAS and RDS. The comparison of the stratified groups of sample was performed by chi-square. *p-value* less than 0.05 was considered significant.

## Results

This study aims to determine the incidence of PH in IUGR neonates with associated pathologies like MAS, RDS and BA.

### Grouping of IUGR neonates and Non-IUGR neonates

Selected neonates were divided into two groups. Group A represents IUGR neonates (n = 96) and group B (reference group) represents non-IUGR neonates (n = 38). Group A constituted 63.5% (n = 61) males and 36.5% (n = 35) females, 51% (n = 49) preterm and 49% (n = 51) term neonates, 72% (n = 69) weighing less than 3^rd^ percentile and 28% (n = 27) weighing less than 10^th^ percentile. While group B constituted 55% (n = 21) females and 45% (n = 17) males, 53% (n = 20) preterm and 47% (n = 18) term neonates, 66% (n = 25) weighing less than 3^rd^ percentile and 34% (n = 13) weighing less than 10^th^ percentile as shown in Table 1.

**Table 1:** Demographic profile of both groups A and B.

Variables	Group A				Group B			
	IUGR neonates (n = 96)	%age	IUGR neonates with PH (n = 32)	%age	Non-IUGR neonates (n = 38)	%age	Non-IUGR neonates with PH (n = 24)	%age
**Gender**								
Male	61	63.5	18	29.5	17	45	13	76.5
Female	35	36.5	14	40	21	55	11	52.3
**Gestational Age (weeks)**								
Preterm	49	51	14	28.5	20	53	12	60
Term	47	49	18	38	18	47	12	67
**Weight Percentile**								
<3	69	72	23	33.3	25	66	18	72
<10	27	28	9	33.3	13	34	6	46
**Diseases**								
MAS	18	18.75	18	100	12	31.5	8	67
BA	33	34.4	11	33.3	14	36.8	7	50
RDS	22	22.9	4	18.1	12	31.5	9	75

### Sub-groups of IUGR neonates (Group A) and non-IUGR neonates (Group B) regarding Gender, GA and WP

The gender distribution of the IUGR neonates showed that more males i.e. 63.5% (n = 61) were included in study as compared to females 36.5% (n = 35) while the reference group consisted of more females 55% (n = 21) as compared to males 45% (n = 17). The GA range was 32 to 40 weeks. In group A, the number of preterm neonates (≤37 weeks of GA) was higher (51%, n = 49) as compared to term neonates (>37 weeks of GA) (49%, n = 47). Similarly, the distribution of GA in group B also depicted more preterm (53%, n = 20) than term (47%, n = 18). When the IUGR neonates were grouped into different categories with respect to WP for GA, it was observed that 72% (n = 69) of neonates were weighed less than the 3^rd^ WP, while those weighing less than 10^th^ WP were 28% (n = 27). Similarly, grouping of non-IUGR neonates showed that 66% (n = 25) of neonates were weighed less than the 3^rd^ WP and 34% (n = 13) were weighed less than 10^th^ WP.

Descriptive statistics was applied to analyze the data which showed that mean age of all IUGR neonates was 3.06 days and the mean age of PH associated IUGR neonates was 2.72 days. Mean birth weight of all IUGR neonates was 1.83 kg. Mean GA of PH associated IUGR neonates was 37.13 weeks as shown in Table 2(a–d).

**Table 2:** Data of neonates (a) stratification of Mean age (days) and weight for GA (kg) in relation to gender (b) descriptive analysis of mean age (days) and birth weight (kg), (c) stratification of Age (days) and GA (weeks) with regards to PH and (d) stratification of different parameters with regards to PH.

(a)								(b)								(c)								
Mean age (days) and weight for GA (kg)								Descriptive statistics								Age (days) and GA (weeks) with PH								
Gender	Age (days)				Birth weight (kg)			Parameters	Minimum	Maximum		Mean		S.D		PH		Age (days)				GA (weeks)		
	Mean		S.D		Mean		S.D											Mean		S.D		Mean		S.D
Male (n = 61)	3.06		1.16		1.83		0.33	Age in days	1.00	8.00		3.0625		1.1681		Yes		2.72		1.14		37.13		2.338
Female (n = 35)	3.13		1.21		1.83		0.33	Birth weight (kg)	1.30	2.40		1.8302		0.3176		No		3.23		1.15		36.67		2.45
**(d)**																								
**Stratification of different parameters with PH**																								
Parameters		Gender				GA (weeks)			WP				MAS				BA				RDS			
PH		Male (n = 61)		Female (n = 35)		Preterm (n = 49)		Term (n = 47)	<3 (n = 69)		<10 (n = 27)		Yes (n = 18)		No (n = 78)		Yes (n = 33)		No (n = 63)		Yes (n = 22)		No (n = 74)	
Yes (n = 32)		18		14		14		18	23		9		18		14		11		21		4		28	
No (n = 64)		43		21		35		29	29		46		0		64		22		42		18		46	
Total		96				96			96				96				96				96			
*p-*value		0.294				0.312			1.0				0.0001				1.0				0.086			

### Incidences of diseases in IUGR neonates (Group A) and non-IUGR neonates (Group B)

**Figure 1:**
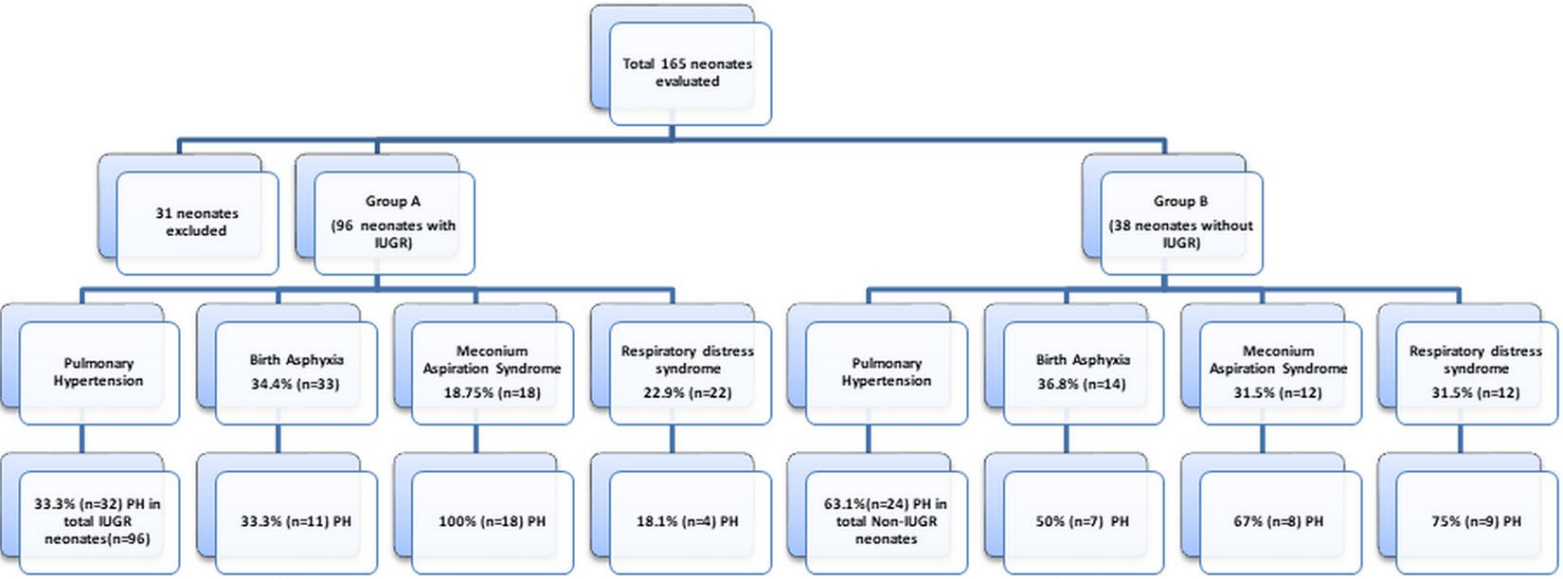
Incidences of diseases (PH, BA, MAS and RDS) in IUGR and non-IUGR neonates.

Since the main outcome variable in this study was PH, it was found that in group A, 33.3% (n = 32) of neonates had PH, while the incidence of PH in group B was 63.1% (n = 24) as shown in Fig. 1. When the incidence of associated diseases was determined in both groups, clinical evidence showed that 18.75%, 34.4% and 22.9% of IUGR neonates and 31.5%, 36.8% and 31.5% of non-IUGR neonates were diagnosed with MAS, BA and RDS, respectively, but the percentage of PH in IUGR neonates accompanying MAS was found to be highest (100%).

### Relationship of gender, GA (weeks) and WP for GA with IUGR neonates (Group A) and non-IUGR neonates (Group B)

#### Relationship of Gender with PH

In Table 1, gender distribution shows that in group A, more females 40% (14/35) suffered from PH compared to males 29.5% (18/61). In group B, more males 76.5% (13/17) suffered from PH compared to females 52.3% (11/21). This showed that occurrence of PH in neonates was not significantly linked to any specific gender. Still we can set forth that males are at increased risk of PH if their growth is restricted during gestational period.

#### Relationship of GA with PH

When GA (weeks) with regards to PH was observed in group A, the results suggested a high incidence of PH in term neonates 38% (18/47) as compared to preterm neonates 28.5% (14/49). Similarly, the results for group B also suggested a high incidence of PH in term neonates 67% (12/18) as compared to preterm neonates 60% (12/20). So we can delineate that premature birth of neonates was not markedly linked with the occurrence of PH in both IUGR as well as non-IUGR neonates. Rather the term neonates were at increased risk of PH.

#### Relationship of WP with PH

When the relationship of WP for GA with PH was observed in group A, it was seen that 33.3% (23/69) neonates (less than 3^rd^ WP) and 33.3% (9/27) neonates (less than 10^th^ WP) suffered from PH, depicting an equal risk of PH in both groups. But when this relationship was observed in non-IUGR neonates, it was revealed that 72% (18/25) were <3 WP neonates having PH and 46% (6/13) were <10 WP. These results are clearly narrating that birth weight (kg) less than 3^rd^ percentile posing an elevated risk of PH in non-IUGR group. But when considering IUGR neonates, the restricted growth in all SGA neonates equally affect both weight percentiles.

As the *p values* for gender (*p* = *0.294*), GA (*p* = *0.312*) and WP (*p* = *1.0)* were not less than 0.05 as shown in Table 2 declaring a non-significant relationship of gender, GA (weeks) and WP for GA with PH in IUGR neonates. When these observations were compared with the reference group, results suggested that gender, GA (weeks) and WP for GA can also cause IUGR independent PH along with IUGR dependent PH. Growth restriction is not a negotiable factor as restricted growth poses profound effect on neonatal health.

### Relationship of MAS, BA and RDS with PH in group A (IUGR neonates) and group B (non-IUGR neonates)

When the stratification of BA, MAS and RDS with regards to PH was done for group A, the results depicted that 100% (18/18) neonates with MAS were diagnosed with PH (*p* = *0.0001*) while in group B, 67% (8/12) neonates with MAS were diagnosed with PH. It was also observed that 33.3% (11/33) IUGR neonates suffering from BA and 18.18% (4/22) with RDS in group A experienced PH but in group B, it was found that 50% (7/14) non-IUGR neonates suffering from BA and 75% (9/12) suffering from RDS were also diagnosed with PH. When Chi-square tests were applied to check the effect modification, it was observed that the MAS was strikingly associated with PH in IUGR neonates as compared to BA and RDS, suggesting that in IUGR neonates the risk of MAS associated PH is higher as compared to non IUGR neonates while BA and RDS are IUGR independent risk factors of PH.

### Severity of PH in IUGR neonates

Stratification of the severity of pulmonary hypertension was performed as mild (20-40 mmHg), moderate (41-60 mmHg) and severe (>60 mmHg). Our results showed that 37.5% neonates had mild, other 37.5% had moderate while 25% had severe PH, suggesting the important role of IUGR in the occurrence of PH as shown in Table 3.

**Table 3:** Incidence of mild, moderate and severe PH in IUGR neonates.

Parameters	Pulmonary Hypertension	Frequency	Percent
No	None	64	100.0
Yes	Mild (26-40 mmHg)	12	37.5
	Moderate (41-60 mmHg)	12	37.5
	Severe (>60)	8	25.0
	Total	32	100.0

## Discussion

**Figure 2:**
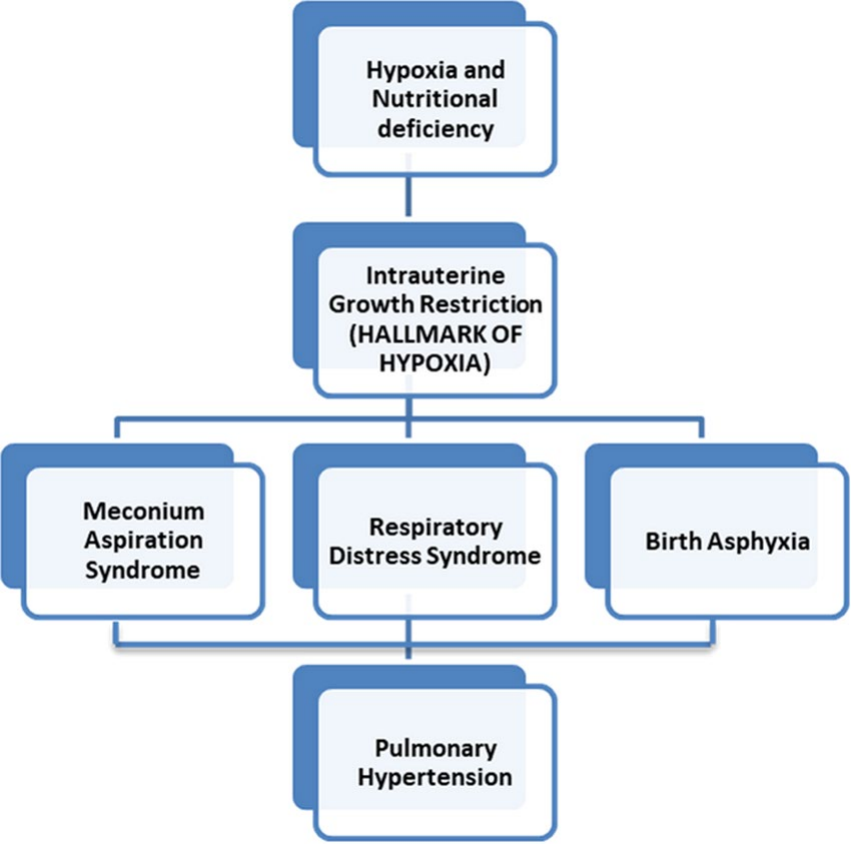
Sequelae of intrauterine growth restriction.

This study is conducted for the first time in Pakistan to describe an association between PH and IUGR in neonates predisposed to various complications such as MAS, BA and RDS. IUGR is a major and silent cause of various morbidities and mortalities in the fetal and neonatal population^26^. Our findings highlight that growth restricted neonates are at increased risk of PH. PH is a complication encountered frequently in IUGR neonates in association with severe intrinsic lung diseases such as MAS, BA and RDS^20,27,28^ as shown in Fig. 2.

Our data suggest a highly significant association of MAS with PH in IUGR neonates (*p* = *0.0001*) but BA, RDS, gender, GA (weeks) and WP as a non-significant risk of PH. In this study, there was a clinical evidence of PH in 33.3% (n = 32) of IUGR neonates as compared to 63.1% (n = 24) of non-IUGR neonates. Our study suggests a varying degree of IUGR associated PH. The documented presence of 37.5% mild, 37.5% moderate and 25% severe PH in IUGR neonates signifies the importance of IUGR in the incidence of PH. Hernández-Díaz, S., *et al*. study showed that male infants were at increased risk of PH as compared to female^29^. Our data showed that the incidence of PH was more in female than male but this risk was non-significant (*p* = *0.294*), suggesting that gender was not a major factor for PH in IUGR neonates. Our study showed that the term neonates were at increased risk of acute episodes of PH rather than preterm, but this incidence of PH in relation with gestational age (weeks) was found to be non-significant (*p* = *0.312*). We studied the incidence of PH in IUGR neonates, but the Hernandez *et al*., included infants in his study. Contrary to our result, Hernandez *et al*., reported that preterm infants were at increased risk of persistent pulmonary hypertension (PPHN) compared to term^29^. A similar result to Hernandez *et al*., was also reported by Bhat *et al*.,^30^. Prematurity with severe IUGR causes respiratory problems in neonates, but the literature has conflicting reports on this aspect^27^. Some studies suggest an advantage to stress from poor growth^31–33^ but some studies have found an increased respiratory morbidity in IUGR neonates^34^. BW is also a non-significant risk factor for PH in IUGR neonates (*p* = *1.0*). Lubchenco *et al*., reported a 3-fold risk for neonatal morbidity in LBW neonates (<10^th^ Percentile) compared to normal BW infants^35^. It was recognized that some LBW babies were the victims of IUGR. In our study, it was found that neonates with BW less than 10^th^ percentile and BW less than 3^rd^ percentile were both vulnerable to PH equally. Contrary to our result, Bhat *et al*., showed that the lower the weight percentile, the higher the vulnerability to neonatal morbidities like RDS, BA and MAS, which then leads to PH^30^. Another related study was conducted on 7 infants. In this study, all infants except one had a weight percentile less than 3, 5 and 10 indicating IUGR, and these IUGR infants were diagnosed with PH^36^.

IUGR is one of the major risk factors in the development of MAS in neonates^37^. This association has also been previously reported in another study^38^. MAS is associated with significant morbidity and mortality rates, often linked to the presence of PH^17^. PH in IUGR neonates with MAS may be due to chronic intrauterine hypoxia^37,39^. Term neonates are more vulnerable to pass meconium in response to such stress than preterm^37^. The key finding of our study was that the MAS was the most common cause of PH. In our study, we examined 18 IUGR neonates accompanying MAS and all of these neonates had an incidence of PH (100%). As fetal distress that is responsible for MAS, thus avoidance of factors resulting in fetal distress and eventually BA might be the key to decrease incidence of PH among the MAS, as observed in study done by Su, B. H. *et al*.^40^.

In the IUGR fetus, perinatal asphyxia is the initial concern^41^. It contributes greatly to perinatal and neonatal mortality. IUGR fetus with intrauterine hypoxia are more prone to asphyxia^21^. In the clinical setting, 34.4% of IUGR neonates were encountered with birth asphyxia, but 33.3% of them manifested with the clinical evidence of PH suggesting BA as a risk factor of PH in IUGR neonates. RDS can occur in IUGR infants with a higher frequency^8^. A marked risk of mortality was found in premature neonates with PH, if they were simultaneously affected with RDS. MAS remains one of the most common causes of neonatal respiratory distress^42^. In our study, a total of 22 neonates had RDS, which gives a total incidence of 22.9%. Of these 18.18% suffered from PH, which showed that RDS also plays some role in the development of PH in IUGR neonates. Premature infants with RDS often requires ventilator assistance^8^ and this mechanical ventilation and oxygen interfere with alveolar and vascular development leading to bronchopulmonary dysplasia (BPD)^43^. Additionally, infants with BPD have increased vascular resistance which leads to PH^44^. Regardless of this mechanism of developing BPD associated PH, IUGR is also an independent risk factor for the development of BPD^45^. In a multicenter investigation by Bose *et al*., 5 fetal growth restriction was found to be an independent prenatal risk factor for BPD^44^. Even IUGR neonates born at term, without prematurity, have worsen respiratory outcomes. IUGR impairs angiogenesis in premature babies, which is essential for the growth of lungs and this IUGR induced impairment in angiogenesis results in abnormal pulmonary vascular development and function, halt alveolarization, decreases pulmonary artery density and ultimately ends in PH^45,46^.

## Limitations

This was a single-center study, which enabled us to collect standardized information on all IUGR neonates, but further investigation into the generalizability of our findings is warranted. Because our sample size was small, we were unable to fully assess the interaction effects of the common covariates.

## Conclusion

Our study leads to a conclusion that restricted growth inside the uterus enhances the risk of PH in neonates. MAS is a IUGR dependent risk factor of PH in neonates. The documented presence of PH demands further investigations about the potential risk factors involved in the development of PH in IUGR neonates.
